# Photothermal Hyperthermia Suppresses Liver Tumor Growth Via Hippo Signaling Pathway-Dependent Inhibition of Cell Proliferation and Induction of Apoptosis

**DOI:** 10.1186/s12575-025-00282-5

**Published:** 2025-06-17

**Authors:** Jian Li, Yuanhua Qin, Yingying Yang, Hang Chen, Mengjuan Li, Yadi Liu, Bingjie Liu, Jingli Shang, Yu Zhang, Tao Han, Yuhan Hu, Feng Ren

**Affiliations:** 1 Department of Pathology, the First Affiliated Hospital of Xinxiang Medical University, Xinxiang, 453003 China; 2 School of Basic Medical Sciences, Xinxiang Medical University, Xinxiang, 453003 China; 3 Department of Gynecology, the First Affiliated Hospital of Xinxiang Medical University, Xinxiang, 453003 China; 4 Henan International Joint Laboratory of Immunity and Targeted Therapy for Liver-Intestinal Tumors, Xinxiang, 453003 China; 5 Henan Research Center for Engineering Technology in Digestive Tract Tumor Immune Digital Decoding and Cell Therapy, Xinxiang, 453003 China

**Keywords:** Hepatocellular carcinoma, Hyperthermia, Hippo pathway, YAP, Cancer therapy

## Abstract

Hepatocellular carcinoma (HCC) ranks as the third most common malignant tumor globally. Although hyperthermia has shown promise as a non-invasive treatment for tumors, its specific mechanisms and impact on HCC remain underexplored. This study aims to investigate the effects of hyperthermia on HCC proliferation and apoptosis and to elucidate the role of the Hippo signaling pathway in these processes. Huh7 and HepG2 HCC cells were cultured at various temperatures (37 °C, 40 °C, 43 °C, and 46 °C), with 37 °C as the control. Cell proliferation, apoptosis, and cell cycle distribution were assessed via CCK-8 assays and flow cytometry. A subcutaneous xenograft model in nude mice, treated with indocyanine green (ICG) and near-infrared (NIR) irradiation to achieve local hyperthermia, was used to evaluate in vivo tumor growth. RNA-seq and KEGG pathway analyses identified differentially expressed genes, and Western blotting and immunofluorescence were used to confirm the involvement of the Hippo signaling pathway. Hyperthermia at 43 °C significantly inhibited Huh7 and HepG2 cell proliferation and induced apoptosis, accompanied by cell cycle arrest. In vivo, local hyperthermia reduced tumor volume and weight in the ICG + NIR-treated group. RNA-seq and KEGG analyses revealed that the Hippo signaling pathway was activated under hyperthermic conditions, with YAP expression and nuclear translocation markedly downregulated. Further experiments showed that YAP overexpression mitigated hyperthermia-induced effects on cell proliferation and apoptosis, underscoring the role of the Hippo pathway. These findings demonstrate that hyperthermia inhibits HCC growth by regulating the Hippo signaling pathway, reducing cell proliferation, and promoting apoptosis. This study highlights the potential of hyperthermia as an effective therapeutic approach for HCC, with implications for developing targeted hyperthermic therapies.

**Supplementary Information:**

The online version contains supplementary material available at 10.1186/s12575-025-00282-5.

## Introduction

Hepatocellular carcinoma (HCC) accounts for approximately 80% of primary liver cancers and represents the most common pathological subtype. In 2022, there were estimated 377,700 new cases and 316,500 deaths due to liver cancer in China alone, ranking as the fourth most prevalent cancer and the second leading cause of cancer-related mortality in the country [1]. Despite advances in systemic therapies, the 5-year survival rate for HCC remains a dismal 18% [2] This high mortality rate underscores the urgent need for new therapeutic strategies and molecular targets to improve HCC outcomes.

Hyperthermia, a treatment modality that elevates tumor tissue temperature, has emerged as a promising approach in cancer therapy, capable of suppressing primary tumor growth with promising outcomes [3]. Hyperthermia can be classified into three main types based on its area of application: local, regional, and systemic [4]. Local hyperthermia is frequently applied in solid tumor treatments, such as squamous cell carcinoma of the head and neck [5]. Regional hyperthermia is utilized for larger treatment areas, including recurrent ovarian cancer via intraperitoneal thermochemotherapy and metastatic esophageal squamous cell carcinoma through regional thermoradiotherapy [6]. Systemic hyperthermia, often combined with other therapies, targets advanced metastatic cancers to improve therapeutic efficacy [7, 8]. Despite its extensive use as an adjuvant therapy, the molecular mechanisms by which hyperthermia exerts its effects are only now becoming better understood.

Recent studies reveal that hyperthermia can induce apoptosis and inhibit cell proliferation in colorectal cancer through miR-34a and p53 upregulation [9]. Similarly, in non-small cell lung carcinoma (NSCLC), hyperthermia promotes caspase-3-dependent apoptosis through the ATM-Chk2-Cdc25C pathway and enhances the expression of p21 and Bax mRNA in A549 cells, inhibiting cell growth [10].These findings indicate hyperthermia’s potential as an effective anticancer therapy with distinct molecular mechanisms. However, the specific mechanisms through which hyperthermia affects HCC remain unclear, highlighting the need to investigate its antitumor effects and underlying pathways in HCC.

The Hippo signaling pathway, initially identified in Drosophila melanogaster, is a crucial regulator of cellular processes, including proliferation, apoptosis, differentiation, and organ size [11, 12]. Abnormal activation of this pathway has been implicated in multiple cancers, notably HCC [13, 14]. Key downstream effectors of the Hippo pathway, Yes-associated protein (YAP) and transcriptional coactivator with PDZ-binding motif (TAZ), are often overexpressed in HCC, where they contribute significantly to tumor initiation, progression, and recurrence [15]. Dysregulated YAP/TAZ activity is associated with liver development, regeneration, and carcinogenesis [16]. In HCC cells, YAP inhibition has been shown to reduce cell proliferation and migration [17]. Several molecules modulate YAP signaling in HCC, such as α-actinin-1, which promotes cell proliferation by reducing YAP phosphorylation, and miR-375, which inhibits cell growth and invasion through YAP suppression [18]. Furthermore, the Hippo pathway interacts with several other pathways, including Wnt, Notch, and TGF-β, playing an essential role in HCC pathogenesis [19].

Given these insights, exploring the interaction between the Hippo pathway and thermal stimulation could provide a theoretical foundation for hyperthermia’s therapeutic effects in HCC, potentially enhancing anti-tumor efficacy via Hippo pathway modulation. In this study, we investigated the effects of hyperthermia on HCC cells through in vitro and in vivo experiments. Our findings demonstrate that hyperthermia effectively inhibits HCC cell proliferation and tumor growth by modulating the Hippo signaling pathway, suggesting its potential as a therapeutic option for HCC.

## Materials and methods

### Cell Culture and Stable Cell Line Construction

HCC cell lines, Huh7 and HepG2, were obtained from the Cell Bank of the Chinese Academy of Sciences Committee (Shanghai, China). The cells were cultured in DMEM supplemented with 10% fetal bovine serum (FBS) at 37 °C with 5% CO_2_. All cell lines were routinely tested for mycoplasma, and the results were consistently negative.

Lentivirus vectors for YAP overexpression were purchased from Genechem Co., LTD. (Shanghai, China). The target cells (2 × 10^5^) were infected with 1 × 10^6^ lentivirus transducing units in the presence of polybrene (1 µg/ml) and subsequently selected with puromycin (2 µg/ml) for approximately 5 days to establish stable YAP overexpression cell lines.

### Cell Proliferation and Colony Formation

Cell proliferation was assessed using the cell counting kit-8 (CCK8) assay. Briefly, 2 × 10^3^ cells were seeded into each well of 96-well plates in triplicate, and incubated at 37 °C with 5% CO_2_. Prior to detection at the indicated times, they were incubated at 37 °C, 40 °C, 43 °C, and 46 °C for 1 h, respectively. 5 µl of CCK-8 buffer (DOJINDO, Japan) was added to each well, and the cells were incubated for 1 h at 37 °C. Subsequently, the absorbance of each well was measured at 450 nm using a Microplate Auto-reader (Bio-Rad, Hercules, CA, USA).

For colony formation assays, cells were plated in 6-well plates (300 cells/well) and cultured for approximately 2 weeks until the appearance of cell colonies. The colonies were fixed and stained with hematoxylin, and those containing more than 50 cells were counted for further statistical analysis. Each experiment was performed thrice.

### Flow Cytometry

A total of 1 × 10^6^ cells, after indicated treatments, were collected by trypsinization and centrifugation, and fixed with 70% cold ethanol. After centrifugation, the cells were incubated with 0.5 ml of propidium iodide (PI) staining buffer containing 200 mg/ml RNaseA and 50 µg/ml PI (Beyotime Biotechnology Co., Ltd, China) at 37 °C for 30 min in the dark. Cell cycle distribution was analyzed by FACScan cytometry (Becton-Dickinson, San Jose, CA, USA).

Cell apoptosis was analyzed using flow cytometry and an Annexin-V-FITC Apoptosis Detection kit following the manufacturer’s instructions (Beyotime Biotechnology Co, China). After hyperthermia, cells were harvested and washed twice with cold PBS. A total of 1 × 10^5^ cells were resuspended in 200 µl of binding buffer supplemented with 10 µl Annexin-V FITC and 5 µl PI, and incubated for 10 min in the dark before analyzed by FACScan cytometry (Becton-Dickinson, San Jose, CA, USA).

### ICG and its Cytotoxicity

The ICG (Indocyanine green) powder (Pulsion Medical Systems, Munich, Germany) was dissolved in sterile saline solution and diluted to 1 mg/ml. The photothermal performance for each of the aqueous solutions of the formulation with different ICG concentrations (0, 50, 100, 200, 400 and 600 µg/ml) was measured after 808 nm laser irradiation (1 W/cm^2^) for a period of 600 s. The cytotoxicity of ICG was detected by using CCK8 assay.

### Animal Experiments

4–6 weeks old BALB/c female nude mice were purchased from the Center of Laboratory Animal Science of Beijing Weitong Lihua (Beijing, China). All animal experiments were conducted according to the National Institutes of Health (NIH) Guidelines for Laboratory Animal Care and approved by the Institutional Animal Care and Use Committee of Xinxiang Medical University. Xenograft tumors were generated by subcutaneous injection of HCC cells. When the tumor reached approximately 80 mm^3^, the nude mice were randomly divided into 3 groups: saline (NC), saline and exposure to near-infrared laser (NIR), and treatment with ICG and exposure to near-infrared laser (ICG + NIR). Each mouse received a single intravenous injection of ICG solution at a dose of 4 mg/kg body weight via the tail vein.

A NIR laser system (GentleLASE, Candela, Wayland, MA, USA) emitting 808 nm light was utilized. The diameter of the laser beam was 8 mm, with a pulse time of 3 ms and a laser radiant exposure of 70 J/cm^2^. The ICG fluorescence intensity was induced and monitored in anesthetized mice using the Kodak imaging system (Carestream Health Molecular Imaging, New Haven, CT, US). The fluorescence intensity was continuously recorded by capturing images of the mice before and 15 min after the administration of ICG. The tumor volume was measured using a caliper every 3 days over the course of 21 days and calculated using the formula: 1/2 × (length × width^2^) [20]. On day 21, the mice were euthanized and their major organs, along with tumors, were excised. The specimens were then fixed in 4% formalin, processed for immunohistochemical staining, and examined for pathological features.

### MRNA Array and KEGG Pathway Annotation

Total RNA was extracted from HepG2 cells, after cultured at 37 °C and 43 °C for 1 h, using RNAiso Plus reagent (Takara, Kusatsu, Shiga, Japan). RNA degradation and contamination were monitored on 1% agarose gels. RNA purity was checked using a NanoPhotometer^®^ spectrophotometer (IMPLEN, CA, USA) and RNA integrity was assessed using a RNA Nano 6000 Assay Kit of the Bioanalyzer 2100 system (Agilent Technologies, CA, USA). A total of 1 µg RNA per sample was used as input material for RNA sample preparations. RNA-seq and bioinformatics were performed at the Beijing Novogene Institute (Novogene, Beijing, China). Sequencing libraries were generated using the NEBNext^®^ Ultra™ RNA Library Prep Kit for Illumina^®^ (NEB, USA) following the manufacturer’s recommendations, and index codes were added to attribute sequences to each sample.

The differentially expressed genes (DEGs) were identified using threshold values of ≥ 2- and ≤ − 2-fold change and an FDR significance level of < 5%. Kyoto Encyclopedia of Genes and Genomes (KEGG) pathway enrichment analyses of DEGs were performed using the web-based tool DAVID 6.8 (https://david.ncifcrf.gov). The results were visualized in a plot by using ggplot2 in the R software environment.

### Western Blotting

Total proteins from cells were lysed on ice in lysis buffer (KeyGen Biotech, China) and were quantified using a bicinchoninic acid (BCA) protein assay (KeyGen Biotech, China). The NE-PER™ Nuclear and Cytoplasmic Extraction Reageants Kit (Thermo Fisher Scientific) was utilized to isolate nuclear and cytosolic fractions. The proteins were separated on 8–12% SDS-polyacrylamide gels and transferred onto polyvinylidene fluoride membrane (PVDF) (Millipore, Billerica, MA, USA). Then, the membranes were blocked in TBST solution with 5% non-fat milk and incubated at 4 °C overnight with the primary antibodies.

All the antibodies used in this study were from ProteinTech Group (Chicago, IL, USA) listed as follows: Anti-Bax (the dilution is 1:1000); Anti-Bcl-2 (the dilution is 1:500); Anti-caspase-3 P17/P19 (the dilution is 1:200); Anti-P21 (the dilution is 1:1000); Anti-P27 (the dilution is 1:500), Anti-cyclin D1 (the dilution is 1:500); Anti-YAP (the dilution is 1:1000); Anti-tubulin (the dilution is 1:1000). Membranes were washed and then incubated in secondary antibodies peroxidase-conjugated AffiniPure Goat Anti-Mouse IgG or Goat Anti-Rabbit IgG (ABclonal Biotech Co, Woburn, MA, USA). The protein bands were visualized by FDbio-Femto ECL Kit (Fdbio, Hangzhou, Zhejiang Province, China).

### Immunofluorescence Analysis

The cells were incubated in petri dishes at 43 °C for 1 h prior to fixation at 4 °C for 15 min with 4% paraformaldehyde. Then, after being blocked with normal goat serum for 30 min, the cells were incubated with primary antibodies anti-YAP (1: 200) at 4 °C overnight, after which they were incubated with Alexa Fluor 594-conjugated goat anti-rabbit IgG (H + L) (Proteintech, Chicago, IL) for 2 h at room temperature. Subsequently, DAPI was used to stain cell nuclei. Then, the stained cells were mounted with 80% glycerol/PBS for subsequent examination by a laser-scanning confocal microscope (FV1000, Olympus, Japan) using FV10-ASW 4.0 viewer software (Olympus, Japan).

### Statistical Analysis

Data were analyzed using the Statistical Package for the Social Sciences version 19.0 software (SPSS Inc., Chicago, IL, USA). Quantitative data are presented as the mean ± standard deviation of at least three independent experiments. Student’s t-test (two-tailed) or one-way ANOVA were used to calculate the difference between two groups or more than two groups. *p* < 0.05 was considered to indicate a statistically significant difference.

## Results

### Photothermal Hyperthermia Inhibits HCC Cell Proliferation

**Fig. 1:**
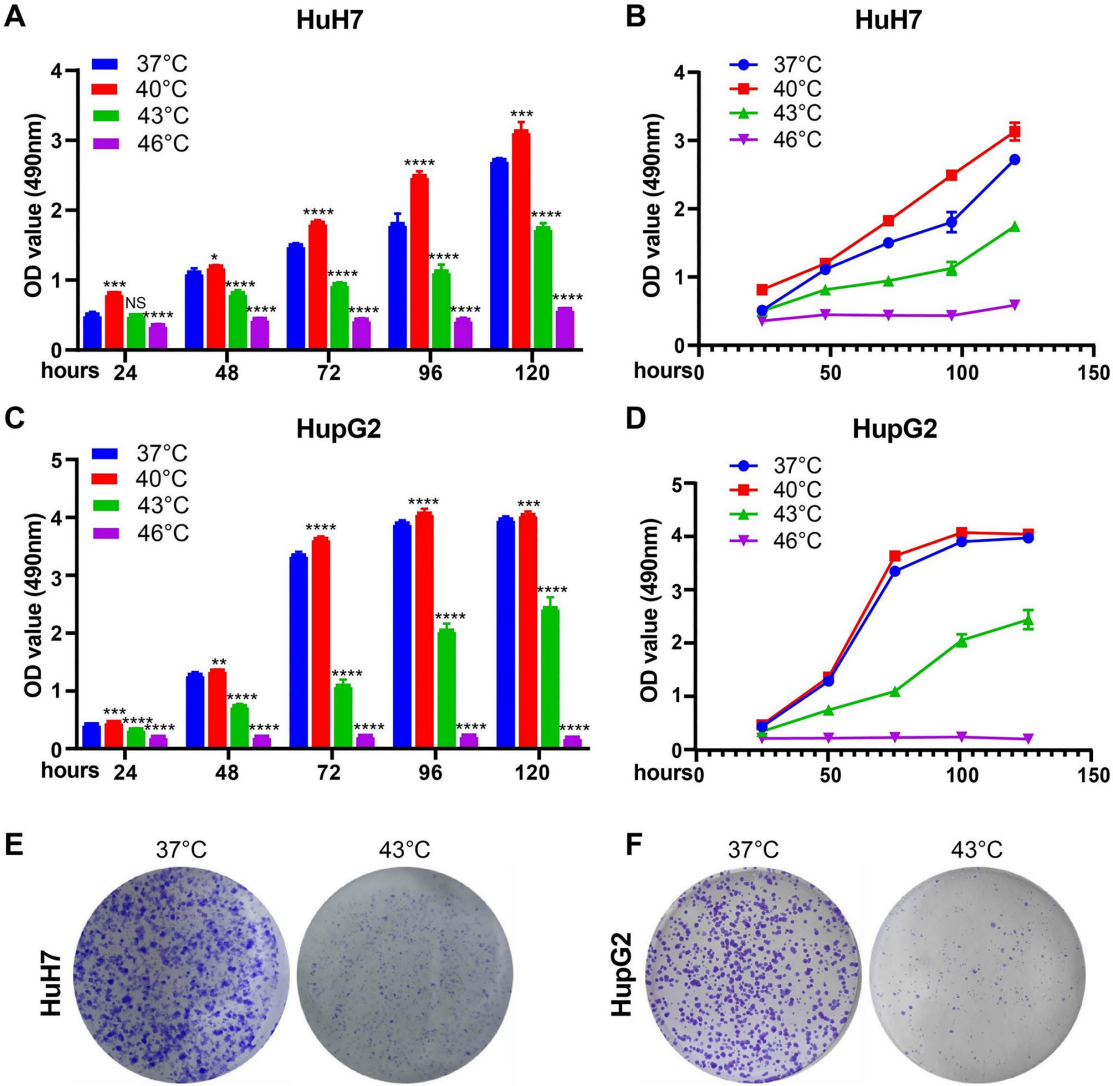
Photothermal hyperthermia inhibited proliferation of Huh7 and HepG2 cells *in vitro.* (**A-D**) cells were treated at different temperatures (37 ℃, 40 ℃, 43 ℃ and 46 ℃). Cell proliferation was evaluated by CCK-8 assay. Data represent mean ± SD of quadruplicate wells. *P < 0.05, **P < 0.01, ***P < 0.001, ****P < 0.0001. (**E** and **F**) Representative images showing the colony forming ability of cells after cultured at 37 °C and 43 ℃

Taking into account the external temperature that the human body can tolerate, we cultivated Huh7 and HepG2 cells at 37 °C, 40 °C, 43 °C and 46 °C, with 37 °C serving as the control. The CCK-8 assay was initially conducted to determine the effects of hyperthermia on HCC cell proliferation. The results indicated that the proliferation of both Huh7 and HepG2 cells was marginally enhanced at 40 °C, but was significantly decreased at 43–46 °C compared to that at 37 °C (Fig. 1A-D). The number of colonies was also significantly reduced in Huh7 and HepG2 cells cultured at 43 °C (Fig. 1E-F). Although cell proliferation was most substantially reduced at 46 °C, subsequent experiments were conducted at 43 °C to avoid potential adverse effects associated with higher temperatures.

### Hyperthermia Promotes HCC Cell Apoptosis and Attests Cell Cycle

**Fig. 2:**
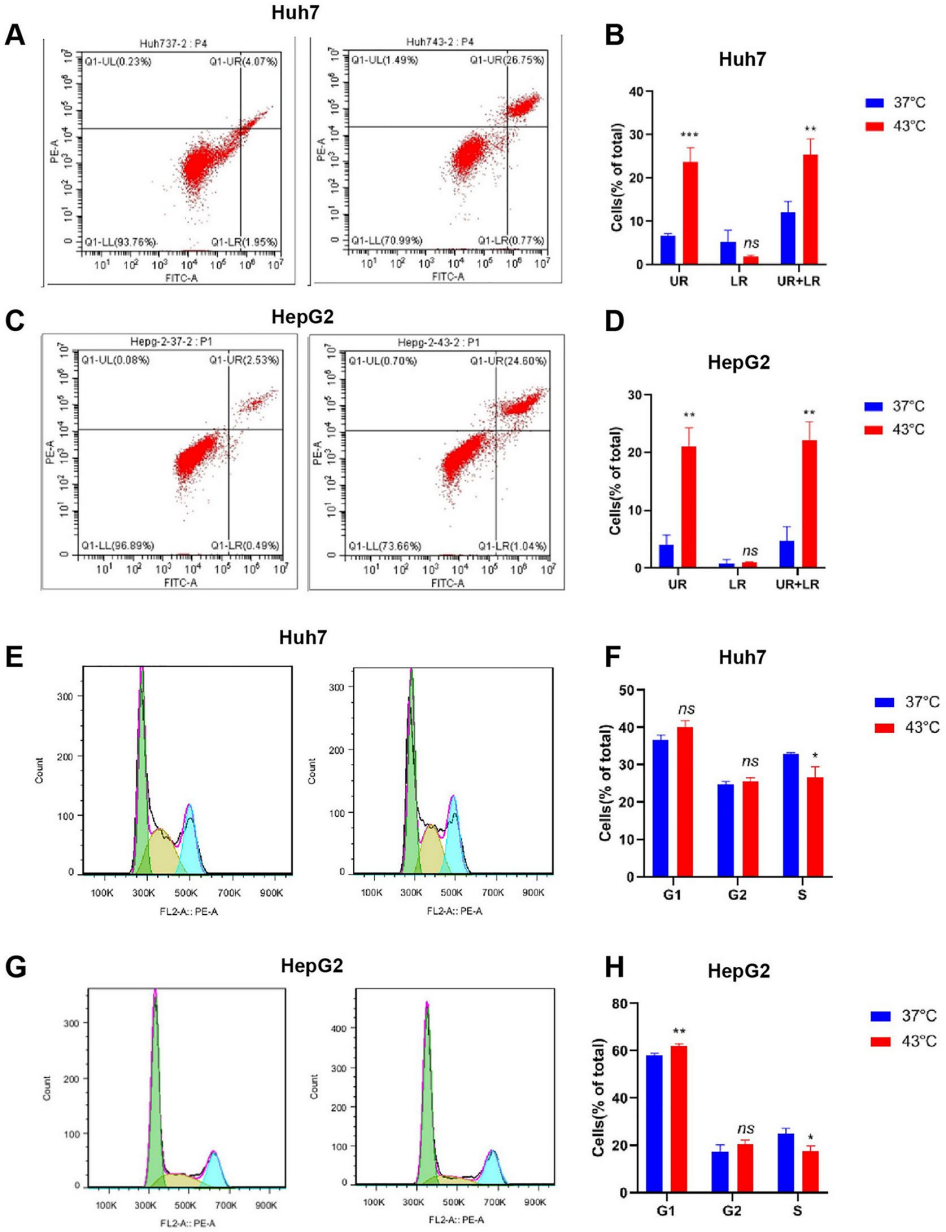
Photothermal hyperthermia inhibited cell proliferation and promoted apoptosis of Huh7 and HepG2 cells in vitro. (**A** and **C**) Analysis of cell apoptosis using flow cytometry in combination with Annexin V/PI double staining. (**B** and **D**) The percentage of cells in early and late apoptosis and total apoptotic cells. Data were from three independent experiments. (**E** and **G**) Analysis of cell cycle using flow cytometry in combination with PI double staining. (**F** and **H**) The percentage of cells in different cell cycle phrases. Data were from three independent experiments. *P < 0.05, **P < 0.01, ***P < 0.001

HCC cells were cultured at 43 °C for 48 h, and the subpopulations of live cells, as well as cells in early and late apoptosis, were analyzed by flow cytometry. The results indicated that, compared with cells cultured at 37 °C, the percentage of apoptotic cells increased from 4.0 to 26.8% for Huh7 cells (*p* < 0.05; Fig. 2A-B). Similar findings were observed in HepG2 cells, with the percentage of apoptotic cells rising from 2.5 to 24.6% (*p* < 0.05; Fig. 2C-D). The distribution of HCC cells in different cell cycle phases was also examined using flow cytometry. The percentage of Huh7 cells in the S phase decreased from 32.9% in cells cultured at 37 °C to 26.7% in cells cultured at 43 °C (*p* < 0.05; Fig. 2E-F). Similarly, the percentage of HepG2 cells in the S phase dropped from 24.9% in cells cultured at 37 °C to 17.9% in cells cultured at 43 °C (*p* < 0.05; Fig. 2G-H). Additionally, the percentage of HepG2 cells in the G1 phase increased from 58.3% in cells cultured at 37 °C to 62.2% in cells cultured at 43 °C (*p* < 0.05; Fig. 2G-H). These results suggest that hyperthermia inhibits HCC cell proliferation by inducing apoptosis and arresting cell cycle progression.

### Photothermal Hyperthermia Inhibits HCC Cell Proliferation in Vivo

**Fig. 3:**
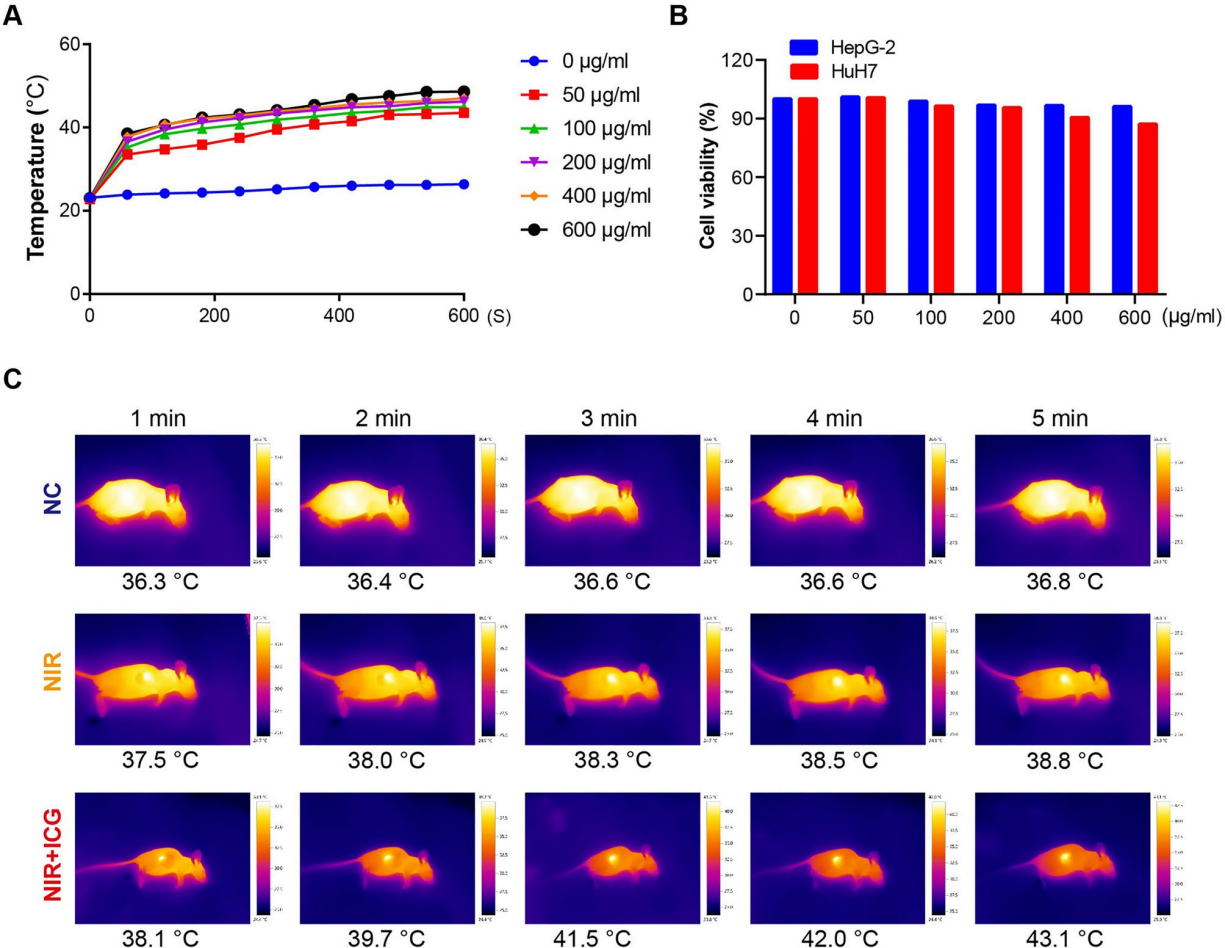
ICG has good heating effect and low cytotoxicity. (**A**) Temperature change curve of ICG solution with different solubility under near infrared irradiation (NIR) of 808 nm, 1 W/cm^2^ for 600 s. (**B**) The cytotoxicity of different concentrations of ICG solutions to liver cancer cells. (**C**) Representative images showing near infrared thermography of mice

**Fig. 4:**
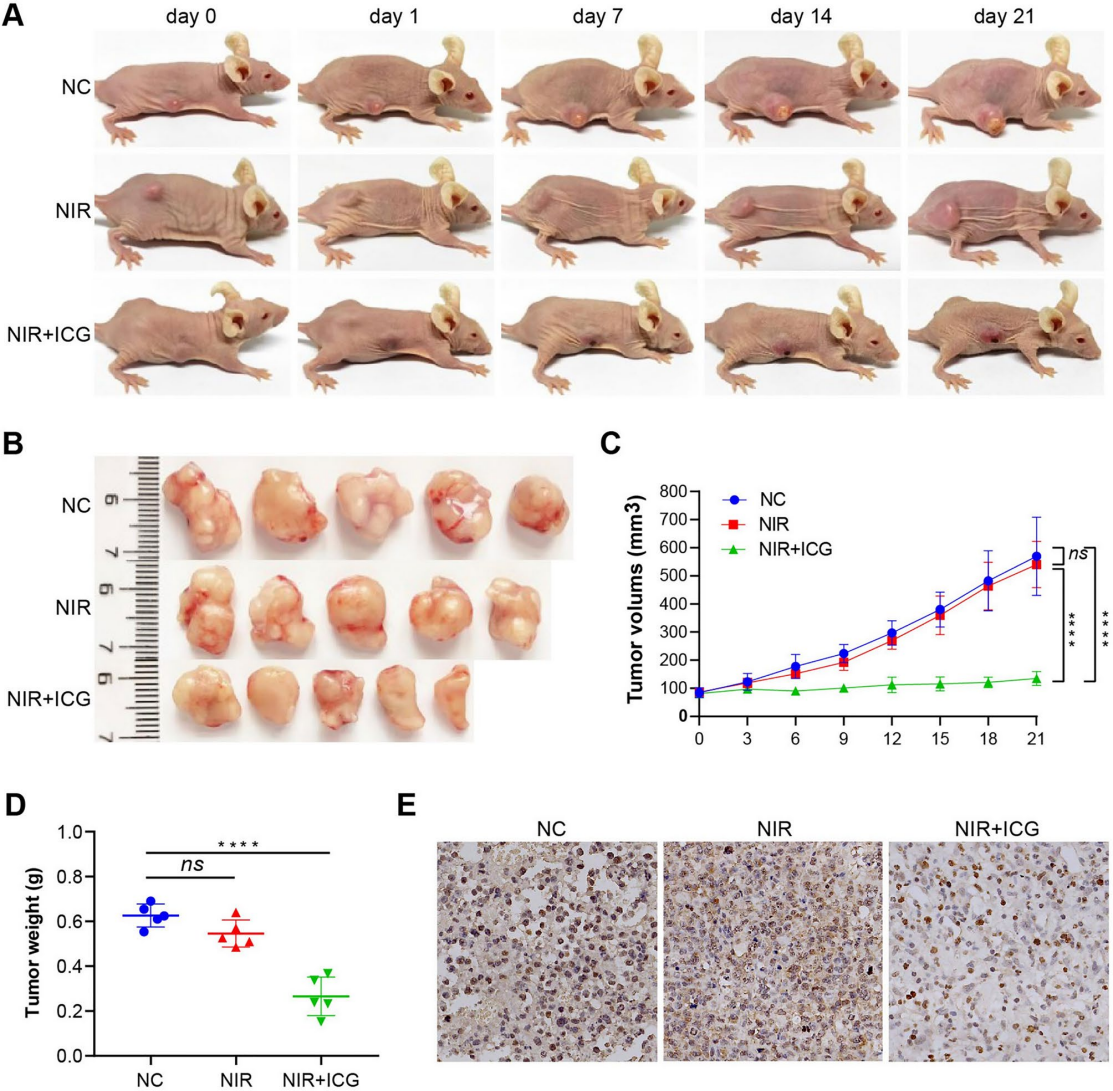
Hyperthermia inhibited liver cancer growth in mice. (**A**) Representative photographs showing HepG2 tumor-bearing nude mice during 21 day treatment. (**B**) Representative photographs showing tumors excised from mice after indicated treatments. (**C**) The changes in tumor volume in HepG2 tumor-bearing mice over indicated times. (**D**) The tumor weights excised from HepG2 tumor-bearing mice. (**E**) Representative histopathological and Ki-67 staining images showing tumor growth in mice xenografted with HepG2 cells. scale bar,. ****P < 0.0001

Treatment with ICG at 50 µg/ml or higher concentrations induced a potent heating effect, raising the temperature to 43 °C within 8 min without a significant impact on cell viability in both Huh7 and HepG2 cells (Fig. 3A-B). Hence, we selected 50 µg/ml ICG to investigate the effect of hyperthermia in vivo. When the xenograft tumor in the nude mice reached approximately 80 mm^3^, exposure to NIR elevated the temperature in the tumors, particularly in ICG-treated mice where the temperature rose to 43 °C in 5 min (Fig. 3C). The volume and weight of tumors formed by HCC cells in the mice treated with ICG and NIR were significantly lower than those in the control mice or mice treated with NIR alone (Fig. 4A-D). Consistently, HCC cell proliferation in the mice treated with ICG and NIR, as indicated by Ki-67 staining, was conspicuously slower than in the control mice and mice treated with NIR (Fig. 4E). Collectively, these results decidedly support that local hyperthermia can effectively inhibit HCC cell proliferation and tumor growth in vivo.

### Photothermal Hyperthermia Regulates the Hippo Signaling Pathway

**Fig. 5:**
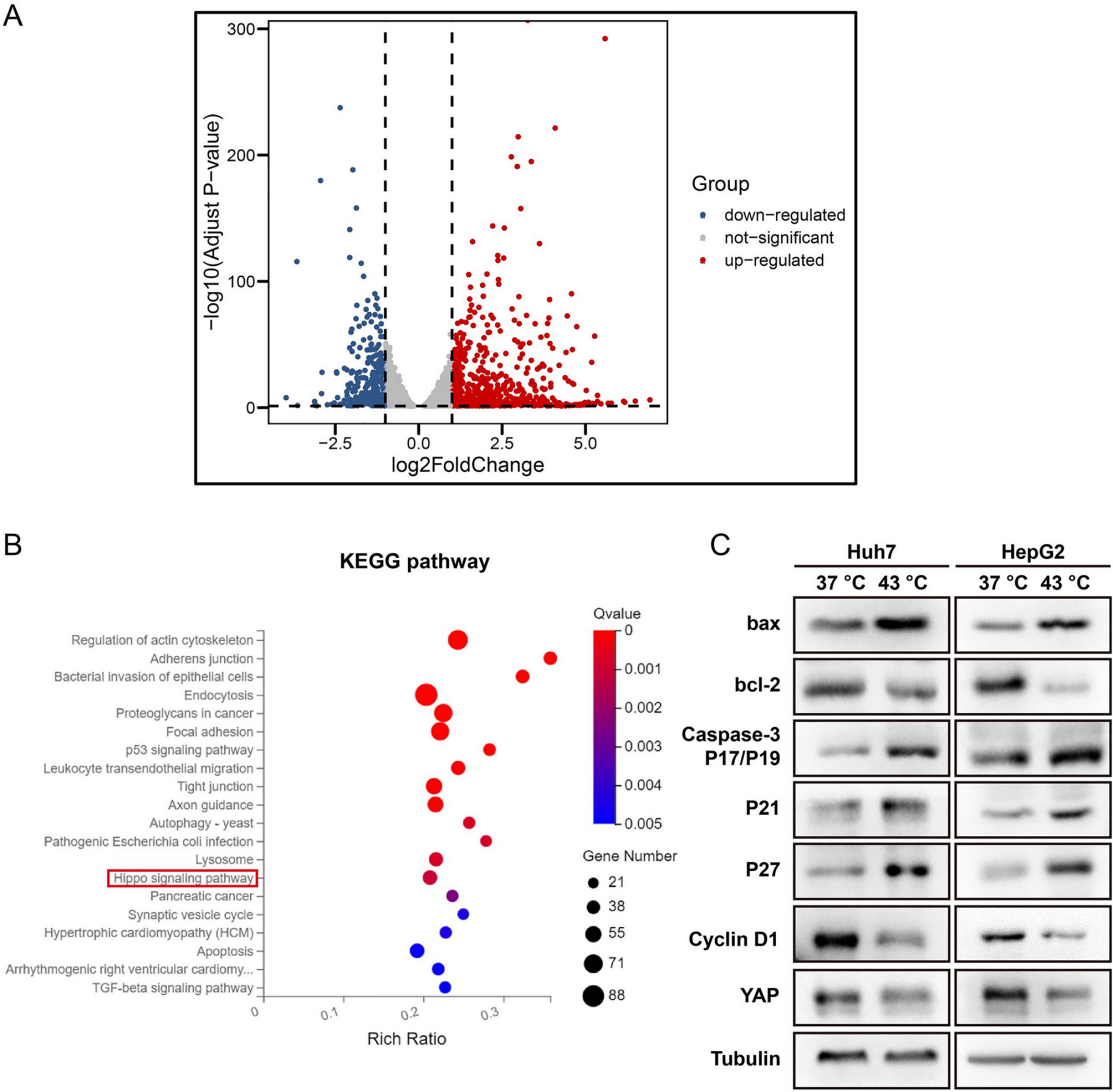
Local hyperthermia regulated the Hippo signaling pathway. (**A**) RNA-seq analysis showed up-regulated and down-regulated gene. (**B**) KEGG pathway analysis of differential gene changes in up-regulated signal pathway. (**C**) Representative blots showing the expression of YAP, cyclin D1, Bcl-2, Bax, caspase-3p17/p19, P21 and P27 in HCC cells under indicated treatments

**Fig. 6:**
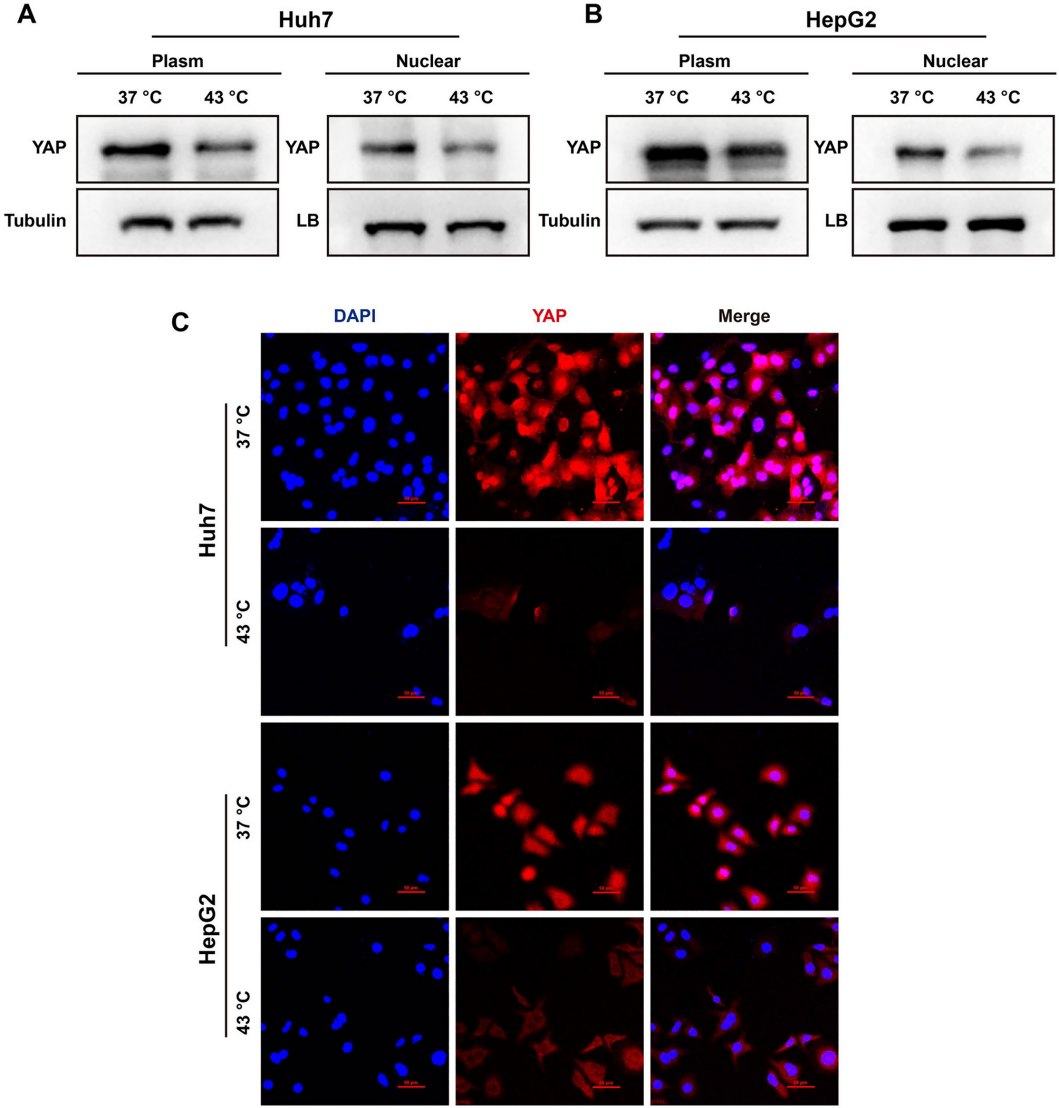
Local hyperthermia inhibited the expression of YAP protein in the nucleus in HCC cells. (**A** and **B**) Representative western blots showing YAP in the nucleus and cytoplasm in HCC cells under indicated conditions. (**C**) Representative immunofluorescence images showing the nuclear localization of YAP protein in HCC cells under indicated conditions

RNA-seq and bioinformatics analyses were conducted to investigate the mechanisms underlying apoptosis and the inhibition of proliferation of HCC cells induced by hyperthermia. RNA-seq disclosed that hyperthermia upregulated 737 genes and downregulated 347 genes (Fig. 5A) in HepG2 cells. The differentially expressed genes (DEGs) were further subjected to KEGG pathway analysis, revealing that the genes encoding molecules in the Hippo signaling pathway were abundant and significantly upregulated in HCC cells cultured at 43 °C compared to those in cells at 37 °C (Fig. 5B). Western blotting was employed to assess the expression of the key molecules in the Hippo signaling pathway, demonstrating that YAP, cyclinD1 and Bcl-2 were significantly down-regulated, while Bax, caspase-3 (P17/P19), P21 and P27 were up-regulated in HCC cells cultured at 43 °C (Fig. 5C). Furthermore, the expression of YAP in the nucleus, examined by Western blotting and immunofluorescent imaging, was reduced in HCC cells cultured at 43 °C compared to that in cells at 37 °C (Fig. 6A-C). Taken together, these results provide compelling evidence to suggest that hyperthermia promotes HCC cell apoptosis and inhibits cell proliferation by regulating the Hippo signaling pathway.

### YAP Can Mitigate the Impacts of High Temperature on the Proliferation and Apoptosis of HCC Cells

**Fig. 7:**
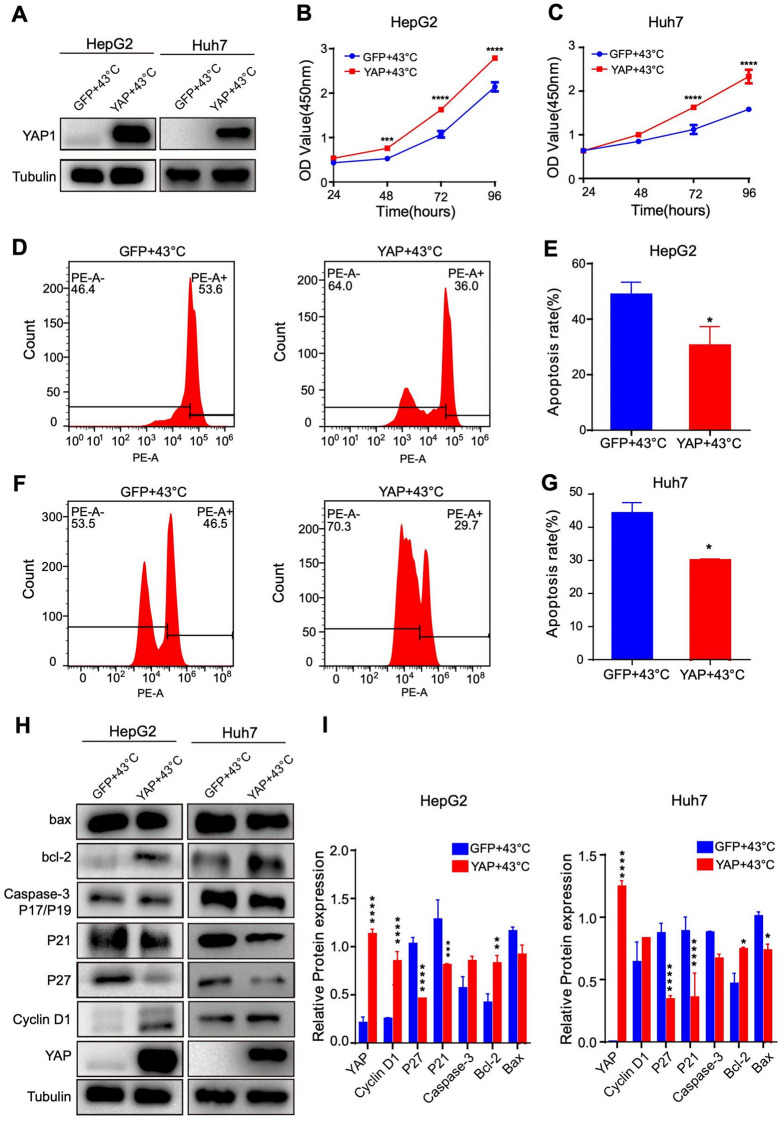
YAP overexpression attenuated hyperthermia-induced apoptosis and cell cycle arrest. (**A**) Representative western blots showing YAP overexpressing in HCC cells. (**B** and **C**) Effects of YAP overexpression on HCC proliferation at 43 °C. (**D**-**G**) Effects of YAP overexpression on apoptosis in HCC cells cultured at 43 °C, detected by Annexin V/PI double staining. (**H** and **I**) Representative western blot and summary of the expression of YAP, cyclin D1, Bcl-2, Bax, caspase-3p17/p19, P21 and P27 in HCC cells without or with YAP overexpression and cultured at 43 °C. *P < 0.05, **P < 0.01, ***P < 0.001, ****P < 0.0001

To further validate the significance of the Hippo signaling pathway in the hyperthermia-induced effects on HCC cells, we overexpressed YAP in HepG2 and Huh7 cells and examined the consequent effects on cell proliferation and apoptosis in cells cultured at 43 °C. YAP overexpression, confirmed by Western blotting (Fig. 7A), significantly enhanced the proliferation of HepG2 and Huh7 cells (Fig. 7B-C). YAP overexpression also significantly decreased the apoptosis of HepG2 and Huh7 cells (Fig. 7D-G). We further investigated by Western blotting the effects of overexpressing YAP on the expression levels of proteins in the aforementioned Hippo signaling pathway. The levels of cyclinD1 and Bcl-2 were significantly higher, and the expression levels of Bax, caspase-3 (P17/P19), P21 and P27 were lower in both Huh7 and HepG2 cells overexpressing YAP than in normal cells (Fig. 7H-I). In contrast, silencing of YAP led to the opposite effects(Fig. S1). These results provide additional evidence to support the importance of the Hippo signaling pathway in HCC cell apoptosis and the inhibition of cell proliferation by hyperthermia.

## Discussion

Current therapeutic strategies for malignant tumors, including surgery, chemotherapy (CT), and radiotherapy (RT), are limited by significant toxicity, systemic side effects, and the risk of developing multidrug resistance, often leading to suboptimal efficacy [21]. Consequently, there is a pressing need for novel, more effective treatments that can extend remission and improve patients’ quality of life.

Hyperthermia has emerged as a non-invasive cancer treatment with considerable potential [22]. It is well established that sustained exposure to temperatures above 42 °C can induce cellular death [23, 24]. At temperatures between 42 °C and 46 °C, hyperthermia disrupts the function of various structural and enzymatic proteins, thereby affecting cell growth, promoting differentiation, and inducing apoptosis [25, 26]. This treatment, which may be used alone or in combination with radiotherapy, chemotherapy, or targeted therapies, acts directly or indirectly to eliminate tumor cells and is widely applied across multiple cancer types [27]. In this study, we provide in vitro and in vivo experimental evidence to illustrate that hyperthermia inhibits the proliferation of HCC cells and induces apoptosis via modulation of the YAP signaling pathway.

Hyperthermia’s anti-tumor effects are closely related to its ability to induce cell cycle arrest and apoptosis. Previous studies have shown that hyperthermia suppresses tumor growth and induces apoptosis by damaging cellular DNA and RNA and/or reducing the synthesis of DNA, RNA, and protein [28, 29]. Mechanistically, heat stress can induce apoptosis via mitochondria in the P53-dependent pathway [30] or cause cell cycle arrest, particularly in the G2/M phase [31]. Consistent with these findings, our study showed that hyperthermia at 43 °C significantly inhibited HepG2 and Huh7 cell proliferation and promoted cell cycle arrest and apoptosis [32] (Fig. 2). It is worthy of mention that some studies have reported that hyperthermia at 43 °C is unable to kill cancer cells, such as human glioma cells T98G and YKG-1, as well as most gastric cancer cell lines [33]. These discrepancies likely arise from variations in tumor cell sensitivity to heat.

The methodology and materials used to induce hyperthermia play an essential role in therapeutic efficacy. Conventional methods, including ultrasound, microwave, radiofrequency, and magnetic induction, each have limitations in clinical settings. Photothermal therapy (PTT) is a newer, less invasive method that offers high specificity, anti-metastatic properties, and fewer adverse effects [34]. PTT is capable of converting light energy into heat under near-infrared (NIR) irradiation [35]. Due to its strong tissue penetration, minimal self-fluorescence, low interference, low cytotoxicity, and high affinity, indocyanine green (ICG) has been approved by the FDA for clinical PTT applications [36, 37]. In our study, we used ICG as a photothermal conversion agent and found that it exhibited high photothermal efficiency and low cytotoxicity under NIR treatment (Fig. 3). The combination of ICG with NIR irradiation effectively suppressed HCC tumor growth in vivo (Fig. 4), highlighting the potential of PTT in HCC treatment. Recent studies also suggest that ICG-containing nanoparticles, used alone or with immune checkpoint inhibitors, may significantly enhance hyperthermia’s anti-tumor efficacy, warranting further investigation into potential combinatory treatments for reducing tumor recurrence [38].

The Hippo signaling pathway, initially identified in Drosophila melanogaster, has been widely studied over the last two decades [12]. This pathway is crucial for regulating cell proliferation, with YAP/TAZ serving as a key component. Elevated YAP/TAZ activity has been reported in several cancers, including HCC, where YAP plays a role in promoting cell proliferation, invasion, and epithelial-mesenchymal transition [39]. Consistent with these observations, our RNA-seq analysis revealed that hyperthermia alters the Hippo signaling pathway in HCC cells (Fig. 5). Additionally, our findings indicate that hyperthermia downregulates YAP expression and reduces its nuclear translocation in HCC cells (Figs. 6 and 7). Evidence suggests that heat stress may induce dephosphorylation and activation of YAP by promoting the ubiquitination and degradation of LATS proteins [40], although further investigation is needed to determine whether this mechanism underlies the observed hyperthermia-induced downregulation of YAP in HCC cells.

In conclusion, our study demonstrates that hyperthermia effectively suppresses HCC tumor growth by inhibiting cell proliferation and inducing apoptosis through modulation of the Hippo signaling pathway. These findings provide compelling evidence supporting hyperthermia as a promising therapeutic approach for liver cancer treatment.

## Electronic Supplementary Material

Below is the link to the electronic supplementary material.

Supplementary Material 1

## Data Availability

The datasets generated during the current study are available from the corresponding author upon reasonable request.

## References

[1] Zheng RS, Chen R, Han BF, Wang SM, Li L, Sun KX, Zeng HM, Wei WW, He J. [Cancer incidence and mortality in China, 2022]. Zhonghua Zhong Liu Za Zhi. 2024;46:221–31. 38468501 10.3760/cma.j.cn112152-20240119-00035

[2] Villanueva A. Hepatocellular carcinoma. N Engl J Med. 2019;380:1450–62. 30970190 10.1056/NEJMra1713263

[3] Mallory M, Gogineni E, Jones GC, Greer L, Simone CB 2nd. Therapeutic hyperthermia: the old, the new, and the upcoming. Crit Rev Oncol Hematol. 2016;97:56–64. 26315383 10.1016/j.critrevonc.2015.08.003

[4] Wust P, Hildebrandt B, Sreenivasa G, Rau B, Gellermann J, Riess H, Felix R, Schlag PM. Hyperthermia in combined treatment of cancer. Lancet Oncol. 2002;3:487–97. 12147435 10.1016/s1470-2045(02)00818-5

[5] Gao S, Zheng M, Ren X, Tang Y, Liang X. Local hyperthermia in head and neck cancer: mechanism, application and advance. Oncotarget. 2016;7:57367–78. 27384678 10.18632/oncotarget.10350 PMC5302995

[6] Sheng L, Ji Y, Wu Q, Du X. Regional hyperthermia combined with radiotherapy for esophageal squamous cell carcinoma with supraclavicular lymph node metastasis. Oncotarget. 2017;8:5339–48. 28029663 10.18632/oncotarget.14148 PMC5354912

[7] Lassche G, Crezee J, Van Herpen CML. Whole-body hyperthermia in combination with systemic therapy in advanced solid malignancies. Crit Rev Oncol Hematol. 2019;139:67–74. 31112883 10.1016/j.critrevonc.2019.04.023

[8] Hurwitz M, Stauffer P. Hyperthermia, radiation and chemotherapy: the role of heat in multidisciplinary cancer care. Semin Oncol. 2014;41:714–29. 25499632 10.1053/j.seminoncol.2014.09.014

[9] Luo Z, Zheng K, Fan Q, Jiang X, Xiong D. Hyperthermia exposure induces apoptosis and inhibits proliferation in HCT116 cells by upregulating miR-34a and causing transcriptional activation of p53. Exp Ther Med. 2017;14:5379–86. 29285066 10.3892/etm.2017.5257 PMC5740804

[10] Wu Z, Wang T, Zhang Y, Zheng Z, Yu S, Jing S, Chen S, Jiang H, Ma S. Anticancer effects of beta-elemene with hyperthermia in lung cancer cells. Exp Ther Med. 2017;13:3153–7. 28588670 10.3892/etm.2017.4350 PMC5450781

[11] Kim W, Jho EH. The history and regulatory mechanism of the Hippo pathway. BMB Rep. 2018;51:106–18. 29397869 10.5483/BMBRep.2018.51.3.022 PMC5882217

[12] Gou J, Lin L, Othmer HG. A model for the Hippo pathway in the Drosophila wing disc. Biophys J. 2018;115:737–47. 30041810 10.1016/j.bpj.2018.07.002 PMC6103738

[13] Thompson BJ. (2020) YAP/TAZ: drivers of tumor growth, metastasis, and resistance to therapy. BioEssays: news and reviews in molecular. Cell Dev Biology 42, e1900162. 10.1002/bies.201900162 32128850

[14] Liu Y, Su P, Zhao W, Li X, Yang X, Fan J, Yang H, Yan C, Mao L, Ding Y, Zhu J, Niu Z, Zhuang T. ZNF213 negatively controls triple negative breast cancer progression via Hippo/YAP signaling. Cancer Sci. 2021;112:2714–27. 33939216 10.1111/cas.14916 PMC8253295

[15] Ma J, Huang X. Research progress in role of Hippo signaling pathway in diagnosis and treatment for hepatocellular carcinoma. Zhong Nan Da Xue Xue Bao Yi Xue Ban. 2021;46:637–43. 34275933 10.11817/j.issn.1672-7347.2021.200243 PMC10930194

[16] Zhang S, Zhou D. Role of the transcriptional coactivators YAP/TAZ in liver cancer. Curr Opin Cell Biol. 2019;61:64–71. 31387016 10.1016/j.ceb.2019.07.006

[17] Zhang T, Zhang J, You X, Liu Q, Du Y, Gao Y, Shan C, Kong G, Wang Y, Yang X, Ye L, Zhang X. Hepatitis B virus X protein modulates oncogene Yes-associated protein by CREB to promote growth of hepatoma cells. Hepatology. 2012;56:2051–9. 22707013 10.1002/hep.25899

[18] Chen Q, Zhou XW, Zhang AJ, He K. ACTN1 supports tumor growth by inhibiting Hippo signaling in hepatocellular carcinoma. J Exp Clin Cancer Res. 2021;40:23. 33413564 10.1186/s13046-020-01821-6 PMC7791991

[19] Kim W, Khan SK, Gvozdenovic-Jeremic J, Kim Y, Dahlman J, Kim H, Park O, Ishitani T, Jho EH, Gao B, Yang Y. Hippo signaling interactions with Wnt/beta-catenin and Notch signaling repress liver tumorigenesis. J Clin Invest. 2017;127:137–52. 27869648 10.1172/JCI88486 PMC5199712

[20] Naito S, von Eschenbach AC, Giavazzi R, Fidler IJ. Growth and metastasis of tumor cells isolated from a human renal cell carcinoma implanted into different organs of nude mice. Cancer Res. 1986;46:4109–15. 3731078

[21] Szwed M, Marczak A. (2024) Application of nanoparticles for magnetic hyperthermia for Cancer Treatment-The current state of knowledge. Cancers (Basel) 16. 10.3390/cancers16061156 PMC10969623 38539491

[22] Bucharskaya AB, Khlebtsov NG, Khlebtsov BN, Maslyakova GN, Navolokin NA, Genin VD, Genina EA, Tuchin VV. (2022) Photothermal and photodynamic therapy of tumors with plasmonic nanoparticles: challenges and prospects. Mater (Basel) 15. 10.3390/ma15041606 PMC8878601 35208145

[23] Bertone V, Barni S, Silvotti MG, Freitas I, Mathe G, Pontiggia P. Hyperthermic effects on the human metastatic liver: a TEM study. Anticancer Res. 1997;17:4713–6. 9494594

[24] Hager ED, Dziambor H, Hohmann D, Gallenbeck D, Stephan M, Popa C. Deep hyperthermia with radiofrequencies in patients with liver metastases from colorectal cancer. Anticancer Res. 1999;19:3403–8. 10629627

[25] Coss RA, Dewey WC, Bamburg JR. Effects of hyperthermia (41.5 degrees) on Chinese hamster ovary cells analyzed in motisis. Cancer Res. 1979;39:1911–8. 445391

[26] Kase K, Hahn GM. Differential heat response of normal and transformed human cells in tissue culture. Nature. 1975;255:228–30. 167282 10.1038/255228a0

[27] Dewey WC, Hopwood LE, Sapareto SA, Gerweck LE. Cellular responses to combinations of hyperthermia and radiation. Radiology. 1977;123:463–74. 322205 10.1148/123.2.463

[28] Kang JK, Kim JC, Shin Y, Han SM, Won WR, Her J, Park JY, Oh KT. Principles and applications of nanomaterial-based hyperthermia in cancer therapy. Arch Pharm Res. 2020;43:46–57. 31993968 10.1007/s12272-020-01206-5

[29] Falkowska-Podstawka M, Wernicki A. Heat shock proteins in health and disease. Pol J Vet Sci. 2003;6:61–70. 12675471

[30] Gu ZT, Li L, Wu F, Zhao P, Yang H, Liu YS, Geng Y, Zhao M, Su L. Heat stress induced apoptosis is triggered by transcription-independent p53, Ca(2+) dyshomeostasis and the subsequent Bax mitochondrial translocation. Sci Rep. 2015;5:11497. 26105784 10.1038/srep11497 PMC4478470

[31] Qi D, Hu Y, Li J, Peng T, Su J, He Y, Ji W. Hyperthermia induces apoptosis of 786-O cells through suppressing Ku80 expression. PLoS ONE. 2015;10:e0122977. 25902193 10.1371/journal.pone.0122977 PMC4406445

[32] Ahmed K, Zaidi SF. Treating cancer with heat: hyperthermia as promising strategy to enhance apoptosis. J Pak Med Assoc. 2013;63:504–8. 23905451

[33] Fukami T, Nakasu S, Baba K, Nakajima M, Matsuda M. Hyperthermia induces translocation of apoptosis-inducing factor (AIF) and apoptosis in human glioma cell lines. J Neurooncol. 2004;70:319–31. 15662973 10.1007/s11060-004-9168-0

[34] Espinosa A, Di Corato R, Kolosnjaj-Tabi J, Flaud P, Pellegrino T, Wilhelm C. Duality of Iron oxide nanoparticles in Cancer therapy: amplification of heating efficiency by magnetic hyperthermia and photothermal bimodal treatment. ACS Nano. 2016;10:2436–46. 26766814 10.1021/acsnano.5b07249

[35] Liu Y, Bhattarai P, Dai Z, Chen X. Photothermal therapy and photoacoustic imaging via nanotheranostics in fighting cancer. Chem Soc Rev. 2019;48:2053–108. 30259015 10.1039/c8cs00618k PMC6437026

[36] Su Z, Ye P, Teng Y, Zhang L, Shu X. Adverse reaction in patients with drug allergy history after simultaneous intravenous fundus fluorescein angiography and indocyanine green angiography. J Ocul Pharmacol Ther. 2012;28:410–3. 22372690 10.1089/jop.2011.0221

[37] Knecht PB, Mantovani A, Herbort CP. Indocyanine green angiography-guided management of Vogt-Koyanagi-Harada disease: differentiation between choroidal scars and active lesions. Int Ophthalmol. 2013;33:571–7. 23277207 10.1007/s10792-012-9692-4

[38] Chen Q, Xu L, Liang C, Wang C, Peng R, Liu Z. Photothermal therapy with immune-adjuvant nanoparticles together with checkpoint Blockade for effective cancer immunotherapy. Nat Commun. 2016;7:13193. 27767031 10.1038/ncomms13193 PMC5078754

[39] Li CL, Li J, Gong SY, Huang M, Li R, Xiong GX, Wang F, Zou QM, Qi Q, Yin XX. Targeting the ILK/YAP axis by LFG-500 blocks epithelial-mesenchymal transition and metastasis. Acta Pharmacol Sin. 2021;42:1847–59. 33879841 10.1038/s41401-021-00655-y PMC8563739

[40] Luo M, Meng Z, Moroishi T, Lin KC, Shen G, Mo F, Shao B, Wei X, Zhang P, Wei Y, Guan KL. Heat stress activates YAP/TAZ to induce the heat shock transcriptome. Nat Cell Biol. 2020;22:1447–59. 33199845 10.1038/s41556-020-00602-9 PMC7757600

