# Supplementary material for: Photothermal Hyperthermia Suppresses Liver Tumor Growth Via Hippo Signaling Pathway-Dependent Inhibition of Cell Proliferation and Induction of Apoptosis
Source: Biol Proced Online. 2025 Jun 17;27:22. doi: 10.1186/s12575-025-00282-5 (PMC12172244; doi:10.1186/s12575-025-00282-5)
Supplement: Supplementary file 1 — Supplementary Material 1 [file 12575_2025_282_MOESM1_ESM.docx]

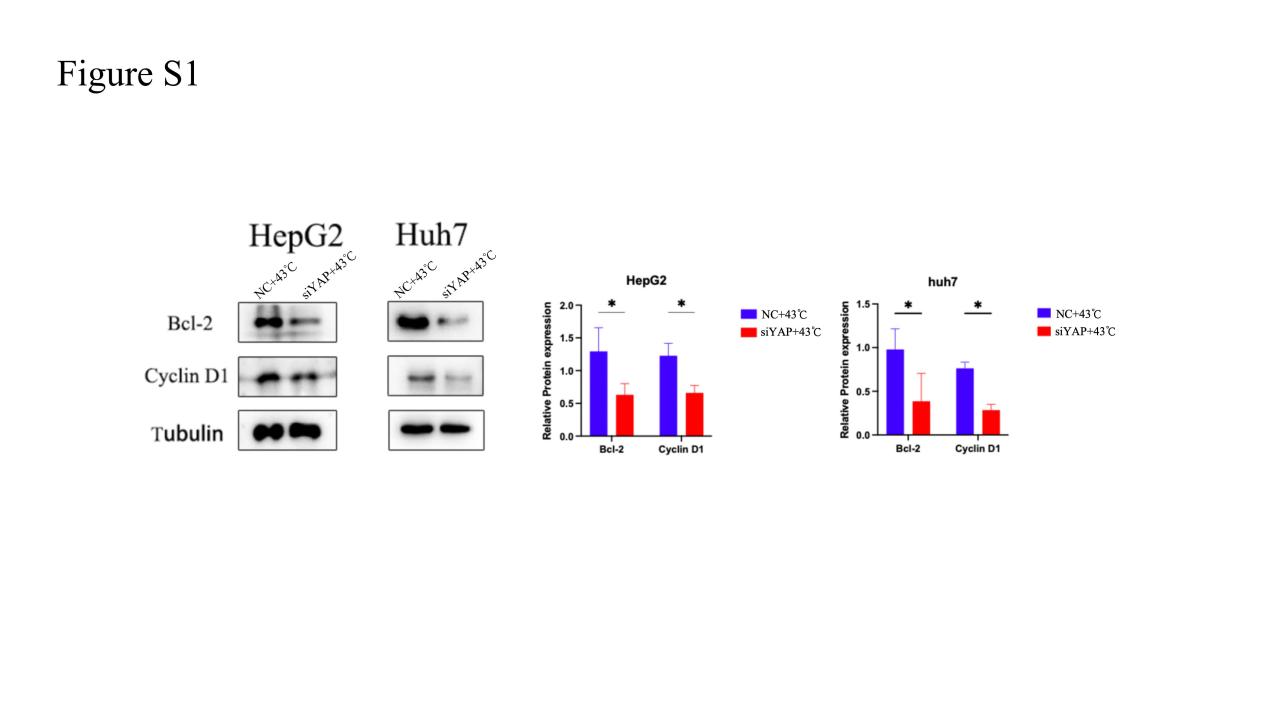


**Figure S1.** Representative western blot and summary of the expression of Bcl-2 and Cyclin D1 in HCC cells without or with YAP inhibition which cultured at 43°C. **P* < 0.05, ***P* < 0.01, ****P* < 0.001.
